# Ginseng oligopeptides protect against irradiation-induced immune dysfunction and intestinal injury

**DOI:** 10.1038/s41598-018-32188-6

**Published:** 2018-09-17

**Authors:** Li-Xia He, Zhao-Feng Zhang, Jian Zhao, Lin Li, Teng Xu, Bin Sun, Jin-Wei Ren, Rui Liu, Qi-He Chen, Jun-Bo Wang, Mohamed M. Salem, Giuseppe Pettinato, Jin-Rong Zhou, Yong Li

**Affiliations:** 1 Department of Nutrition and Food Hygiene, School of Public Health, Peking University, Beijing, 100191 China; 2 Beijing Key Laboratory of Toxicological Research and Risk Assessment for Food Safety, Peking University, Beijing, 100191 China; 3 Department of Surgery, Beth Israel Deaconess Medical Center, Harvard Medical School, Boston, MA 02215 USA; 4 College of Veterinary Medicine, Nanjing Agricultural University, Nanjing, 210095 China; 5 Department of Neurosurgery, Beth Israel Deaconess Medical Center, Harvard Medical School, Boston, MA 02215 USA

## Abstract

Intestinal injury and immune dysfunction are commonly encountered after irradiation therapy. While the curative abilities of ginseng root have been reported in prior studies, there is little known regarding its role in immunoregulation of intestinal repairability in cancer patients treated with irradiation. Our current study aims to closely examine the protective effects of ginseng-derived small molecule oligopeptides (*Panax ginseng* C. A. Mey.) (GOP) against irradiation-induced immune dysfunction and subsequent intestinal injury, using *in vitro* and *in vivo* models. Expectedly, irradiation treatment resulted in increased intestinal permeability along with mucosal injury in both Caco-2 cells and mice, probably due to disruption of the intestinal epithelial barrier, leading to high plasma lipopolysaccharide (LPS) and pro-inflammatory cytokines levels. However, when the cells were treated with GOP, this led to diminished concentration of plasma LPS and cytokines (IL-1 and TNF-α), suggesting its dampening effect on inflammatory and oxidative stress, and potential role in restoring normal baseline intestinal permeability. Moreover, the Caco-2 cells treated with GOP showed high trans-epithelial electrical resistance (TEER) and low FITC-dextran paracellular permeability when compared to the control group. This could be explained by the higher levels of tight junction proteins (ZO-1 and Occludin) expression along with reduced expression of the apoptosis-related proteins (Bax and Caspase-3) noticed in the GOP-treated cells, highlighting its role in preserving intestinal permeability, through prevention of their degradation while maintaining normal levels of expression. Further confirmatory *in vivo* data showed that GOP-treated mice exhibited high concentrations of lymphocytes (CD3^+^, CD4^+^, CD8^+^) in the intestine, to rescue the irradiation-induced damage and restore baseline intestinal integrity. Therefore, we propose that GOP can be used as an adjuvant therapy to attenuate irradiation-induced immune dysfunction and intestinal injury in cancer patients.

## Introduction

The dose of the therapeutic radiation given to patients is usually limited by their tolerance to its numerous side effects. The resultant damage to intestinal mucosa, lymphoid organs (e.g. spleen and lymph nodes) as a consequence to proton irradiation therapy in cancer patients has always been a major limitation for this treatment modality. These side effects can lead to increased risk of infections, especially when radiation is combined with other toxic modalities such as chemotherapy or immunotherapy^1^. Therefore, several strategies have been developed in a trial to combat these adverse effects, in order to preserve the intestinal barrier function, in addition to maximizing the therapeutic benefit from the radiation^2^. Food-derived bioactive peptides, which have low molecular weight and are easily digested and absorbed, have several physiological functions, including antimicrobial, cholesterol-lowering, antioxidant, and cyto- or immunomodulatory activities^3,4^.

Ginseng (*Panax ginseng* C. A. Mey.), traditionally used as a restorative medicine, and been widely used in Chinese communities for thousands of years^5^. The ginseng root has a complex structure including different molecules (e.g. polysaccharides, ginsenosides, peptides and phytosterols); attributing to a its wide range of biological properties including anti-cancer, anti-oxidative^5,6^, anti-diabetic^7^, and immunoregulatory activities^8–10^. In 2002, the Ministry of Health in China issued the “Further Regulating Health Care Product Raw Materials Management Notice”, including ginseng on the “List of Items Used in Health Care Products”. Furthermore, they no longer considered ginseng and its derivatives as restorative medicine, changing its classification as “food”, similar to what happened in developed countries such as the United States, Canada, and Japan.

While previous studies have demonstrated different therapeutic benefits of the whole root of ginseng, there is still no information available regarding its immunoregulatory role and intestinal damage repairability in cancer patients. Therefore, we sought to assess the effect of ginseng oligopeptides on alleviating immune suppression and mitigating the intestinal barrier damage caused by radiation treatment, and to further delineate the underlying regulatory mechanisms of these oligopeptides in mice.

## Results

### Effects of GOP on TEER of Caco-2 cell monolayer

**Figure 1:**
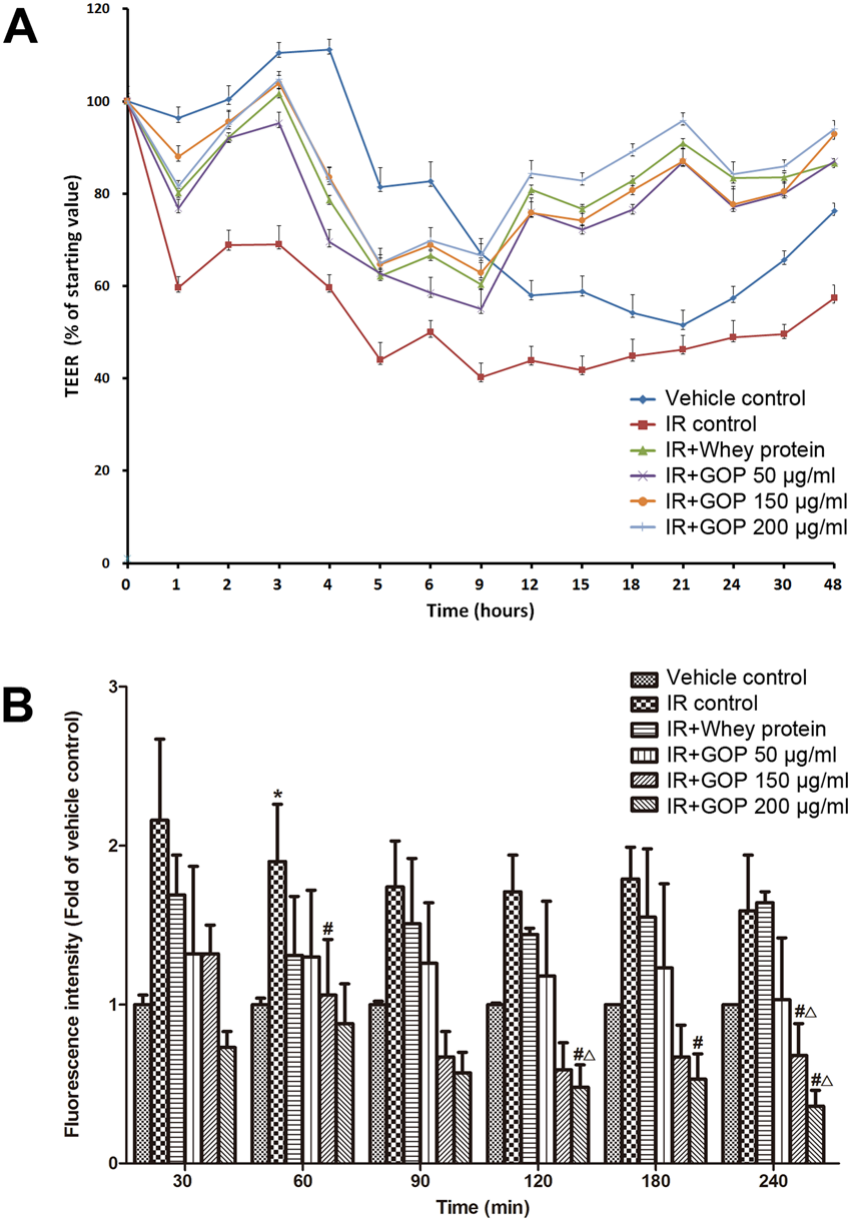
Effects of GOP on the paracellular permeability of Caco-2 cell monolayer after irradiation. (**A**) TEER (% of starting value) at different hours post-irradiation, (**B**) Fluorescence intensity (Fold of vehicle control) for time points after adding FITC-dextran.

TEER of Caco-2 cell monolayers was measured to determine the effect of GOP on the integrity of the epithelial barrier. TEER of Caco-2 layer was monitored for up to 48 hr, and initial measurement was carried out immediately before the radiation exposure. Percentage change of TEER after radiation exposure with respect to the pre-irradiation value was presented. As shown in Fig. 1A, TEER values in all groups decreased at the first-hour post-irradiation, followed by an increase in the following observation time period. At three hours post-irradiation, the transient peak was observed, which can be ascribed to environmental/procedural stress response of the cells to the irradiation. After the transient peak, TEER values went down and tended to be uniform at 5–9 h post-irradiation. Caco-2 cells started to grow back at around 12 h post-irradiation and persist up to 48 h post-irradiation. During all the time points, the TEER values of Caco-2 cell monolayer incubated with whey protein and different concentration of GOP showed significant differences compared to the IR control group incubated only with the vehicle after irradiation. The radio-resistance capacity of Caco-2 cells treated with whey protein and GOP was much higher than the cells without treatments and persisted at similar levels that were comparable with the non-irradiated cells before to reach the 9 h post-irradiation. We observed an increased radio-resistance of the treated cells from 12 h to 48 h post-irradiation to that was higher than the non-irradiated cells.

### Effects of GOP on the paracellular permeability of Caco-2 cell monolayer after irradiation

Gut permeability is one of the primordial functions of the gut epithelium and paracellular permeability of Caco-2 cell monolayer is commonly used as a marker of epithelial integrity. We analyzed the paracellular passage of 4000 Da fluorescein isothiocyanate-dextran (FITC-dextran), which normally cannot penetrate the cellular membrane under physiological conditions. As shown in Fig. 1B, irradiation has led to an increase in permeability compared to the control. A significant effect on epithelial hyper-permeability to FITC-dextran was observed at 60 min, indicating that the cells treated with GOP restored the monolayer integrity. Notably, the monolayer integrity was increased by treatments with 150 and 200 μg/ml GOP (*P* = 0.082 and 0.052, respectively), and the beneficial effects were better than whey protein (*P* = 0.087 and <0.05, respectively) starting from 120 min incubation. At 240 minutes following FITC-dextran administration, cells treated with 150 and 200 μg/ml GOP showed that values significantly higher than the cells treated with whey protein, and without treatments.

### Effects of GOP on body weight (BW) and immune organ index in mice after whole-body irradiation

Thymus and spleen are the two organs that are responsible for the immune system regulation, and they are directly correlated to the number of immune cells circulating in the body, therefore they can be considered important indicators of the immune dysfunction induced by irradiation. The liver is the largest digestive gland in the body and the main metabolic organ, where protein synthesis and metabolism of exogenous and endogenous substrates are performed. A liver damage could lead to a metabolic disorder in the body, such as oxidative stress, reactive carbonyl compounds increase, etc. Therefore, we investigated the treatment effects on liver, spleen, and thymus. Irradiation significantly reduced spleen and thymus indices, as compared to the vehicle control (Table 1). Oral administration of 0.30 g/kg BW GOP improved lightly liver index (*p* = 0.081) and 0.60 g/kg BW increased lightly thymus index (*p* = 0.063) in comparison with the IR + whey protein group.

**Table 1:** Effects of GOP on body weight and immune organ index in mice after whole-body irradiation. The data were presented as the mean ± SD (n = 12), which were analyzed by one-way analysis of variance (ANONA) test, and followed by least-significant difference or Dunnett’s T3 for posthoc test between multiple groups. *p < 0.05 versus vehicle control group, #p < 0.05 versus IR control group, and ^Δ^p < 0.05 versus IR + whey protein group. ^♯^Liver index: Liver weight/body weight; Spleen index: Spleen weight/body weight; Thymus index: Thymus weight/body weight.

Groups	Initial body weight (g)	Final body weight (g)	Liver index (mg/g)^♯^	Spleen index (mg/g)^♯^	Thymus index (mg/g)^♯^
Vehicle control	19.73 ± 1.46	20.69 ± 1.53	37.48 ± 2.94	4.09 ± 0.35	1.64 ± 0.44
IR control	19.33 ± 1.54	19.17 ± 0.65	36.70 ± 2.03	1.47 ± 0.13*	0.75 ± 0.21*
IR + Whey protein	19.45 ± 1.27	19.39 ± 0.97	36.37 ± 3.23	1.55 ± 0.18*	0.69 ± 0.22*
IR + GOP 0.15 g/kg BW	19.10 ± 1.21	19.21 ± 1.52	37.27 ± 3.58	1.48 ± 0.15*	0.76 ± 0.27*
IR + GOP 0.30 g/kg BW	18.90 ± 1.03	19.36 ± 0.46	38.48 ± 2.80	1.54 ± 0.21*	0.70 ± 0.25*
IR + GOP 0.60 g/kg BW	18.97 ± 0.91	19.30 ± 0.77	37.14 ± 2.35	1.48 ± 0.12*	0.94 ± 0.33*

### GOP administration decreases intestinal damage and restores intestinal integrity

**Figure 2:**
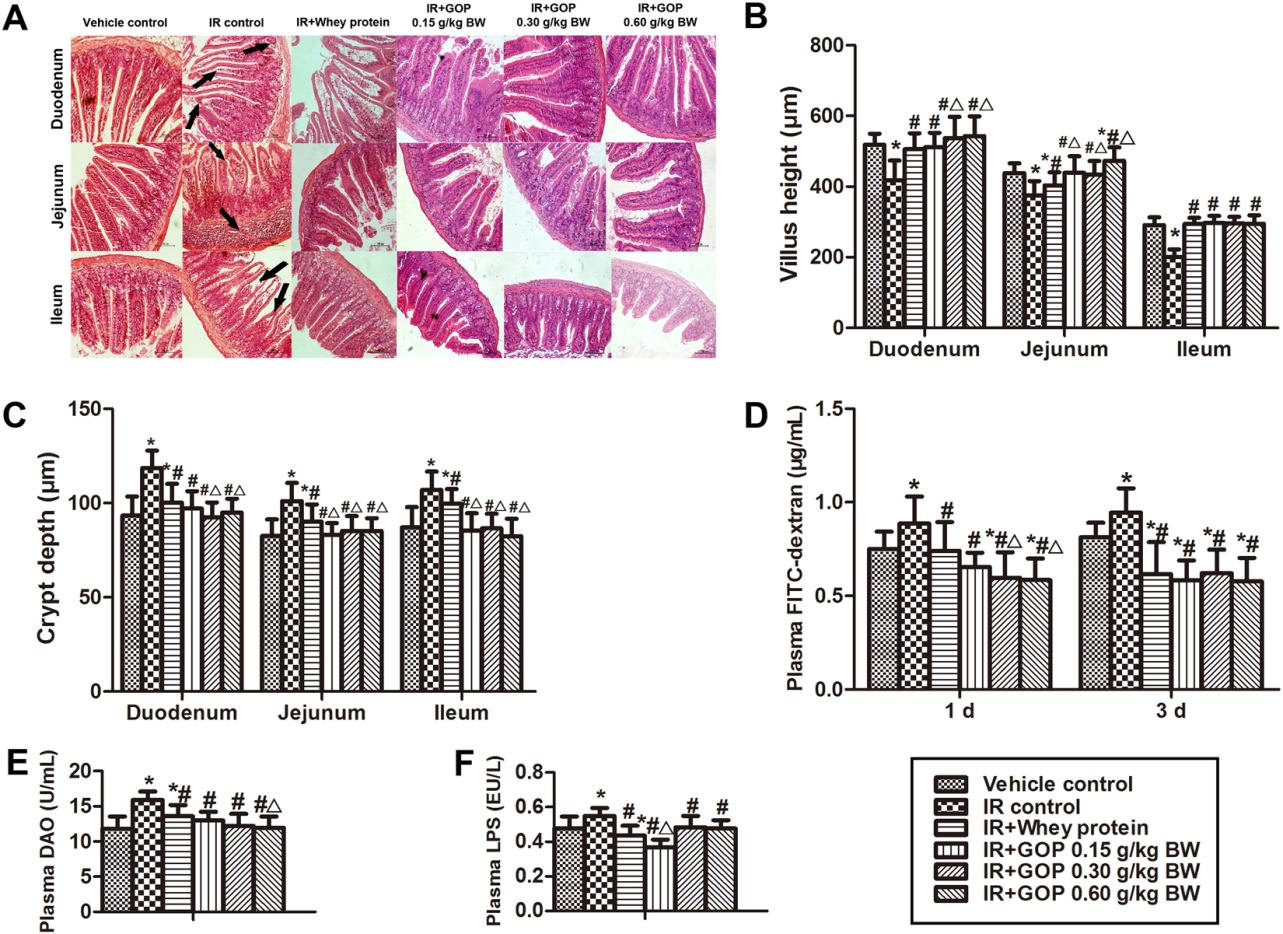
GOP administration decreases intestinal damage and restores the intestinal integrity of irradiated mice. (**A**) Histopathology analysis of intestinal morphology, H&E (magnification × 20), (**B**) Villus height (μm), measured by ImageJ software, (**C**) Crypt depth (μm), measured by ImageJ software, (**D**) Plasma FITC-dextran concentration (μg/mL), measured by FlexStation 2 fluorescence microplate reader, (**E**) Plasma DAO (U/mL), analyzed by ELISA, and (F) Plasma LPS (endotoxin) concentration (EU/L), analyzed by ELISA. Values represented the mean ± SD (n = 10 per group) from a single experiment, which was analyzed by ANOVA test, and followed by least-significant difference for posthoc test between multiple groups. All the measurements were done in duplicates. *p < 0.05 versus vehicle control group, ^#^*p* < 0.05 versus IR control group, and ^Δ^*p* < 0.05 versus IR + whey protein group.

Histological examination of small intestines showed that irradiation caused epithelial atrophy or slough and crypts collapse accompanied by villi shortening and crypts dilating, including marked edema and inflammatory cell infiltration (Fig. 2A). Irradiation shortened villus length (Fig. 2B) and increased crypt depth (Fig. 2C), and GOP administration decreased intestinal damage and restored intestinal integrity by normalizing the villus height and crypt depth (Fig. 2B,C).

### GOP treatment reduces intestinal permeability

Irradiated mice exhibited a higher plasma FITC-dextran concentration as compared to mice in vehicle control group, while GOP and whey protein administration significantly decreased plasma FITC-dextran concentration of irradiated mice at one day and three days after irradiation (Fig. 2D). Moreover, feeding irradiated mice with 0.30 and 0.60 mg/kg BW GOP induced significant reduction in plasma FITC-dextran concentration compared with vehicle control group and IR + whey protein group at one day after irradiation. Therefore, GOP could reduce intestinal damage in a short time as compared to whey protein.

In accordance with the *in vivo* assessment of intestinal permeability, irradiated mice had a significant increase in plasma diamine oxidase (DAO) (Fig. 2E) and endotoxin (lipopolysaccharide, LPS) (Fig. 2F) concentrations compared with vehicle control group. Although reducing plasma DAO and LPS concentrations compared with IR control group, whey protein treatment still increased plasma DAO concentration in comparison with vehicle control group. However, we observed that feeding irradiated mice with GOP decreased plasma DAO and LPS concentrations to the normal levels, further reducing the plasma concentration of LPS. We observed a reduction in plasma DAO concentration of irradiated mice fed 0.30 (*p* = 0.080) and 0.60 mg/kg BW GOP (*p* < 0.05) as compared to IR + whey protein.

### GOP adding rescues irradiation-reduced oxidative stress

**Figure 3:**
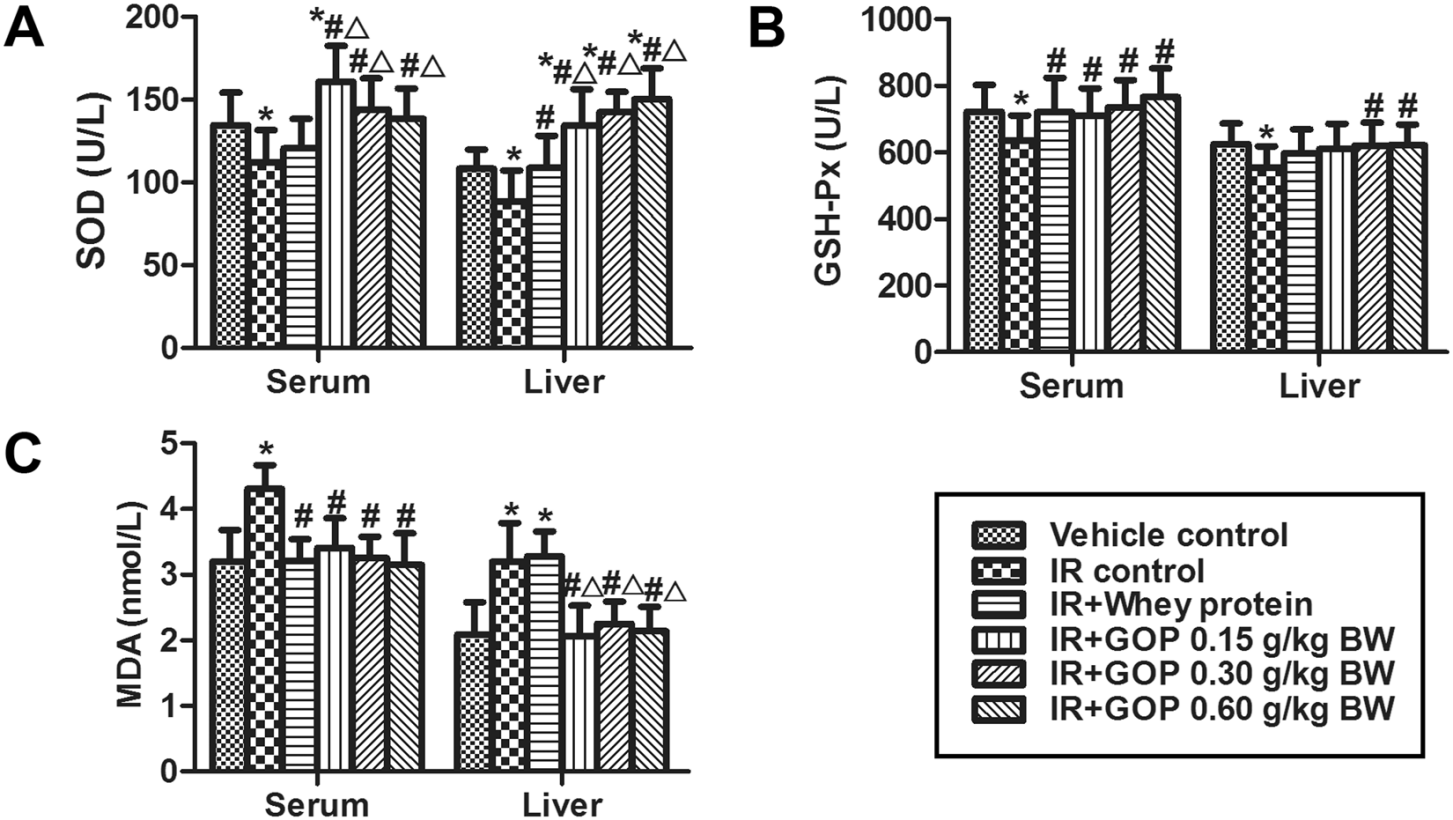
GOP adding rescues irradiation-reduced oxidative stress of mice. (**A**) SOD activity (U/L), (**B**) GSH-Px activity (U/L) and (C) MDA contents (nmol/L) in serum and liver determined by ELISA. Values represented the mean ± SD (n = 12 per group) from a single experiment, which was analyzed by ANOVA test, and followed by least-significant difference for posthoc test between multiple groups. All the measurements were done in duplicates. *p < 0.05 versus vehicle control group, ^#^*p* < 0.05 versus IR control group, and ^△^*p* < 0.05 versus IR + whey protein group.

The generation of free radicals and increased lipid peroxides in tissues is the main reason for cellular injury induced by irradiation exposure. Therefore, we assessed the antioxidant indices in serum and liver of mice. Figure 3 showed that irradiation resulted in a reduction of superoxide dismutase (SOD) and glutathione peroxidase (GSH-Px) activities and the increase of malondialdehyde (MDA) content, while when we added GOP these returned to the normal level. Therefore GOP showed a better ability in improving the SOD activity in serum and liver and decreasing MDA content in liver than the whey protein.

### GOP supplement improves proliferation response of splenic lymphocyte to ConA and LPS, and IL-6 and IL-12 productions in the culture of splenocytes

**Figure 4:**
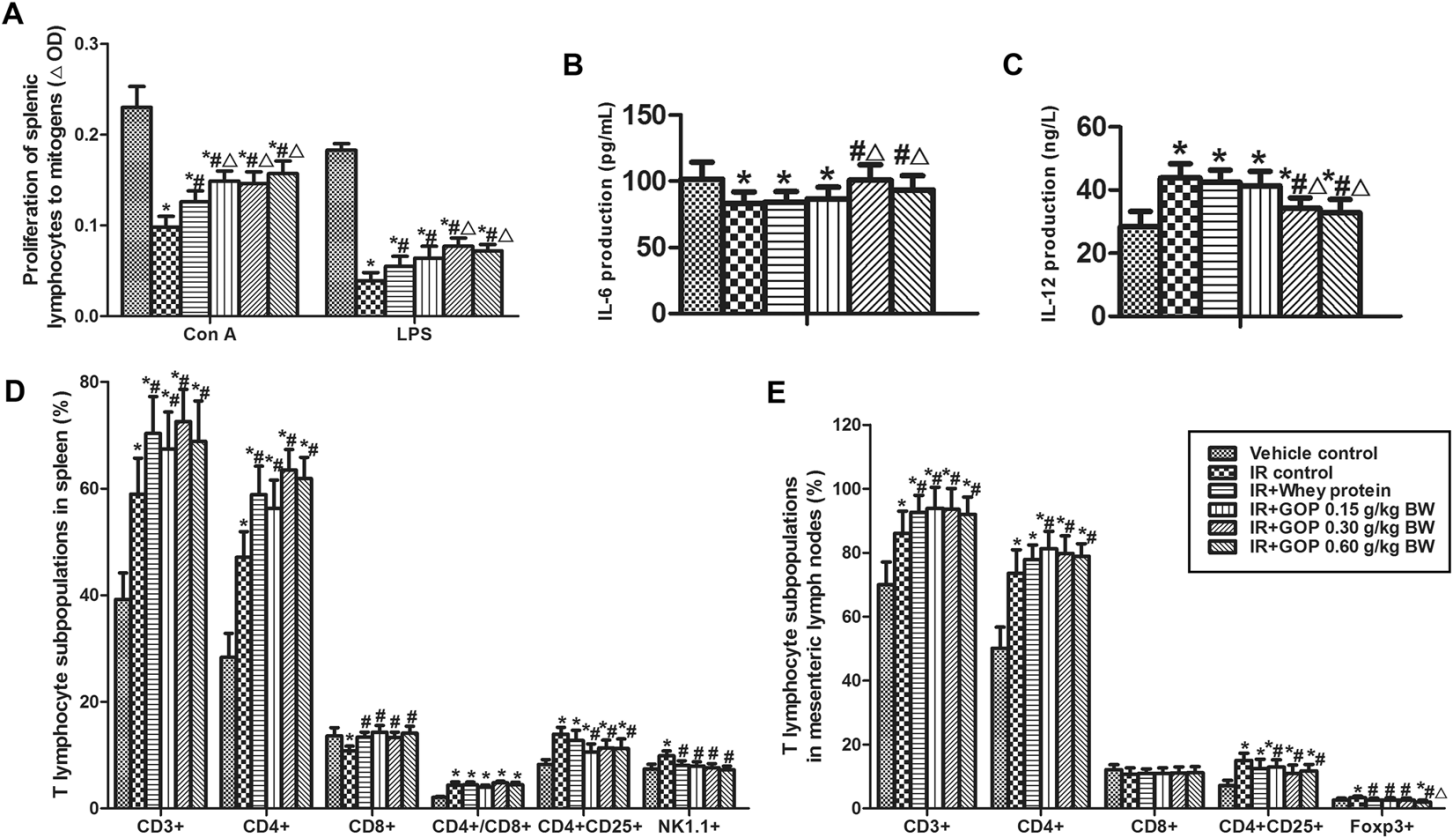
GOP supplement improves proliferation response of splenic lymphocyte, IL-6 and IL-12 productions in the culture of splenocytes of irradiated mice. (**A**) Proliferation of splenic lymphocytes to mitogens (ΔOD), measured by MTT method described in the Materials and Methods, (**B**) IL-6 production in splenocytes (pg/mL), measured by ELISA, (**C**) IL-12 production in splenocytes (ng/L), measured by ELISA, (D) T lymphocyte subpopulations in mesenteric lymph nodes (%), analyzed by flow cytometry, and (E) T lymphocyte subpopulations in spleen (%) analyzed by flow cytometry. Values represented the mean ± SD (n = 12 per group) from a single experiment, which was analyzed by ANOVA test, and followed by least-significant difference for posthoc test between multiple groups. All the measurements were done in duplicates. *p < 0.05 versus vehicle control group, ^#^*p* < 0.05 versus IR control group, and ^△^*p* < 0.05 versus IR + whey protein group.

In contrast with vehicle control group, irradiation reduced the proliferation response of splenic lymphocyte to ConA and LPS, while oral GOP and whey protein administration showed ability in limiting the reduction of splenic lymphocyte proliferation after irradiation. As compared to the IR + whey protein group, GOP 0.30 and 0.60 g/kg BW significantly increased the proliferation response of splenic lymphocyte to ConA and LPS of irradiated mice, and GOP 0.15 g/kg BW had the same trend in the ConA-stimulated proliferation of splenic lymphocyte (Fig. 4A).

The treatment of irradiated mice with GOP 0.30 and 0.60 mg/kg BW significantly lowered the production of IL-12 after irradiation (Fig. 4B), in contrast, improved the irradiation-induced IL-6 inhibition in the culture of splenocytes as compared to IR group and IR + whey protein group (Fig. 4C).

### GOP feeding modulates T lymphocyte subpopulations in spleen and mesenteric lymph nodes

To identify whether GOP may increase the activities of T cells by altering the quantities of T cells or their subpopulations, we conducted a phenotypic analysis of total T cells and T cells subsets in mesenteric lymph nodes (Fig. 4D) and spleen (Fig. 4E).

We found that irradiation resulted in significant increase in the percentages of total T cells (CD3^+^), CD4^+^, CD4^+^/CD8^+^, CD4^+^CD25^+^ and NK1.1^+^, and a significant reduction in the percentage of CD8^+^ in the spleen. Interestingly, GOP supplementation contributed to this trend in the percentages of CD3^+^ and CD4^+^, but weakened the changes of CD8^+^, CD4^+^CD25^+^, and NK1.1^+^, and even made CD8^+^ and NK1.1^+^ return to normal levels, as a result of no significant effect on CD4^+^/CD8^+^. In accordance with the results observed in the spleen studies, irradiation and GOP affected the percentages of CD3^+^, CD4^+^, and CD4^+^CD25^+^ in mesenteric lymph nodes with the same trend, but did not affect significantly the percentage of CD8^+^ although irradiation had a trend in reducing it (*p* = 0.08). Moreover, adding GOP significantly reduced the increase of Foxp3^+^ percentage induced by irradiation, and made it return to normal level. Although GOP did not show more significant effects than whey protein, however, statistical results showed a better trend in those indices (*p* < 0.10).

### GOP administration modulates T lymphocytes in the intestine

**Figure 5:**
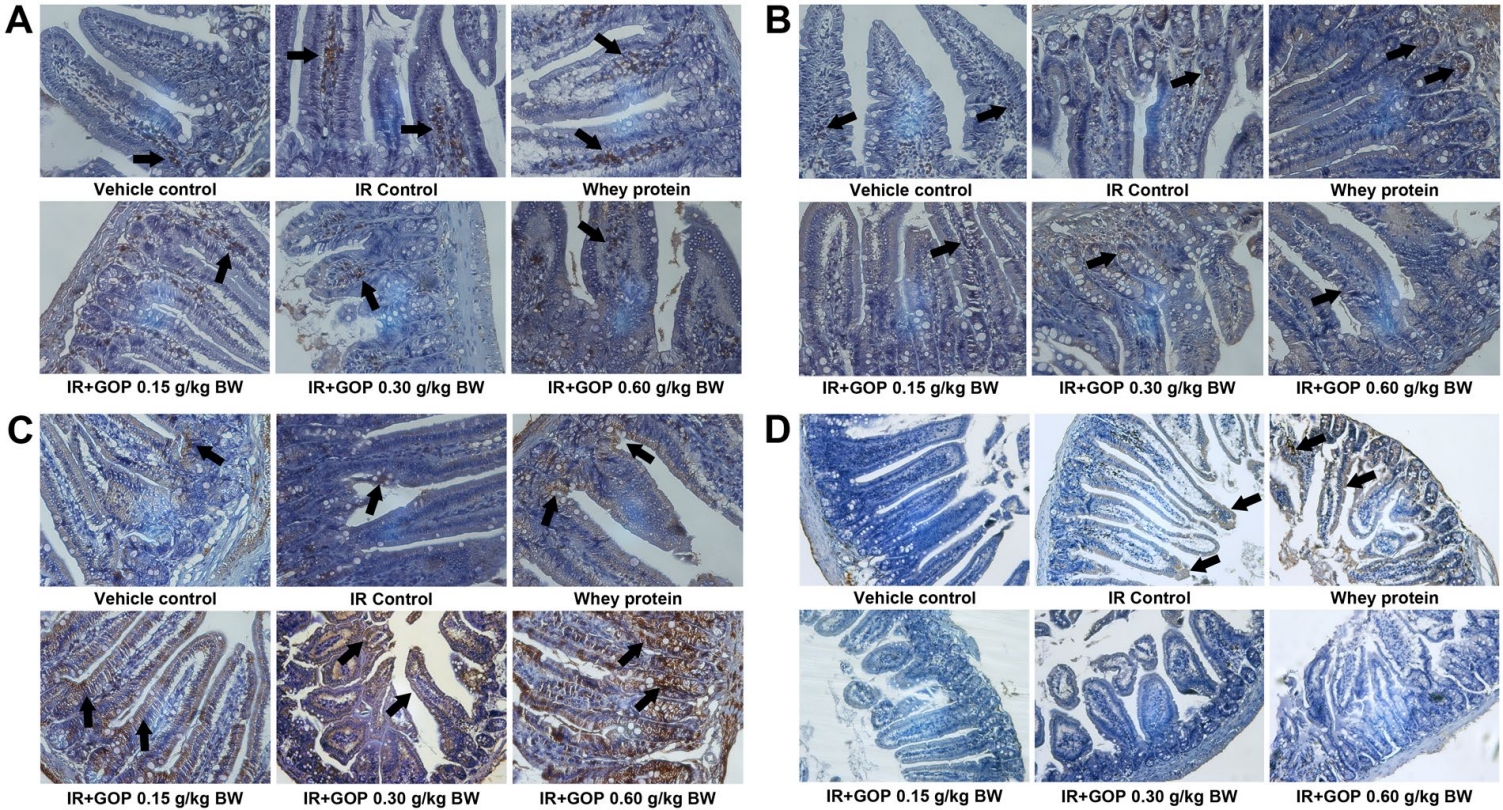
GOP administration modulates T lymphocytes in the intestine. (**A**) CD3^+^ expression, (**B**) CD4^+^ expression, (**C**) CD8^+^ expression and (**D**) CD25^+^ expression. The protein levels were determined by immunohistochemistry.

To identify whether GOP may affects T lymphocytes in the intestine, we performed an immunohistochemistry staining of T lymphocytes subsets in the small intestine coming from irradiated animals previously exposed to all the treatments listed previously. As shown in Fig. 5, irradiated mice exhibited the similar trends in T lymphocytes changes in the small intestine with those in the spleen and mesenteric lymph nodes compared with the mice in the vehicle group. Mice treated with GOP showed increases in CD3^+^, CD4^+^ and CD8^+^ expressions and a decrease in CD25^+^ expression in intestine compared with the mice without GOP treatments.

### GOP administration modulates serum cytokines and immunoglobulins and enhances intestinal SIgA

**Figure 6:**
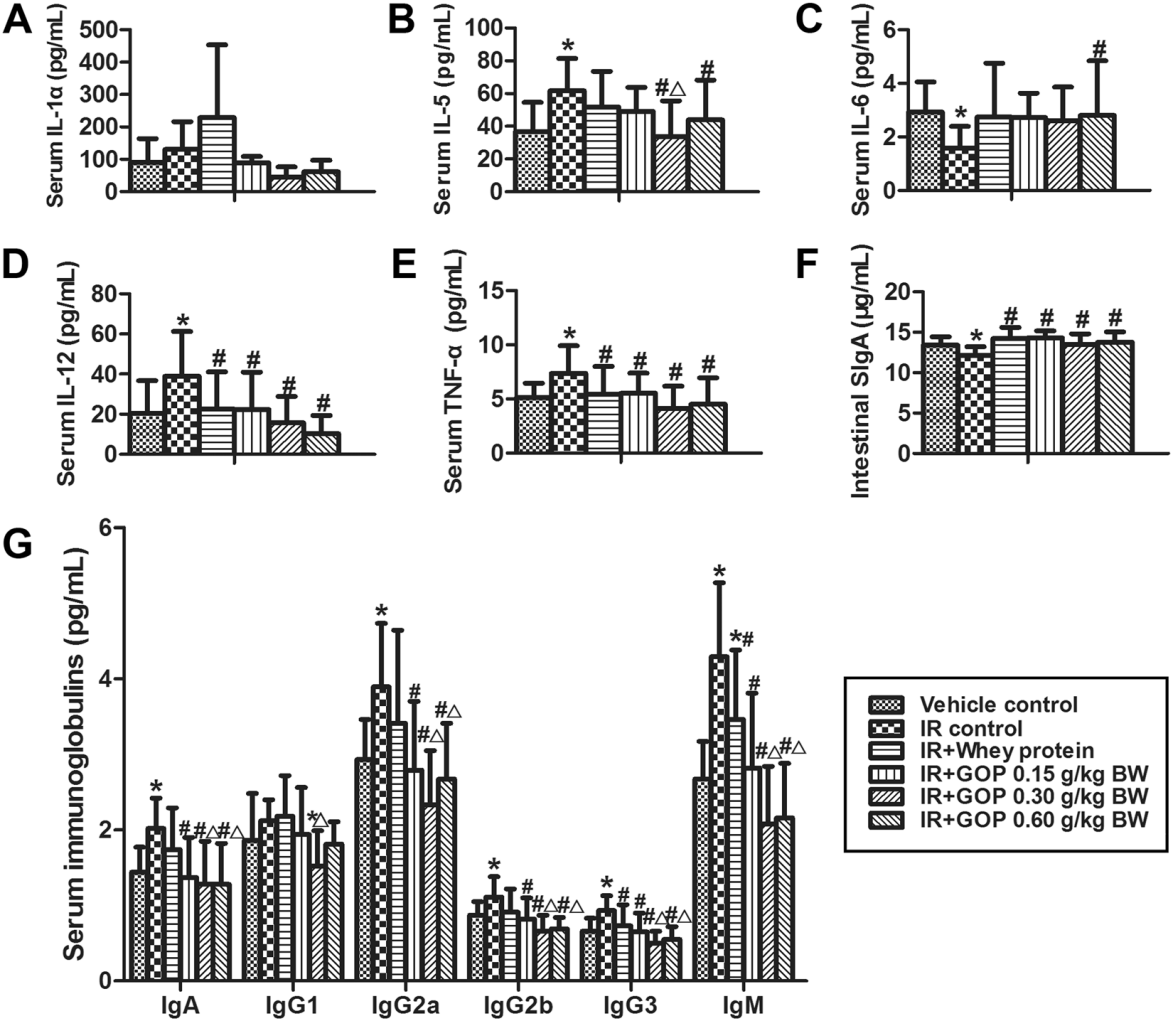
GOP administration modulates cytokine and immunoglobulin concentrations of irradiated mice. (**A**) Serum IL-1α concentration (pg/mL), (**B**) Serum IL-5 concentration (pg/mL), (**C**) Serum IL-6 concentration (pg/mL), (D) Serum IL-12p70 concentration (pg/mL), (**E**) Serum TNF-α concentration (pg/mL), (**F**) Intestinal SIgA concentration (μg/mL) determined by ELISA and (**G**) Serum immunoglobulin concentrations (pg/mL), determined by multiplex sandwich immunoassays. Values represented the mean ± SD (n = 12 per group) from a single experiment, which was analyzed by ANOVA test, and followed by least-significant difference for posthoc test between multiple groups. Serum IL-1α concentration was analyzed by the nonparametric Kruskal-Wallis test was used to determine statistical differences, and pairwise comparisons were used for posthoc test between multiple groups. To decrease the probability of Type 1 error, the adjusted significances were calculated. All the measurements were done in duplicates. *p < 0.05 versus vehicle control group, ^#^*p* < 0.05 versus IR control group, and ^Δ^*p* < 0.05 versus IR + whey protein group.

In this research, irradiated mice treated with GOP 0.30 mg/kg BW showed higher IL-1α concentration in serum compared with vehicle control group (*p* = 0.088) and IR + whey protein group (*p* = 0.074) (Fig. 6A). Furthermore, irradiation caused a significant increase in serum of IL-5, IL-12p70, and TNF-α concentrations, and reduction in the serum of IL-6 concentration (Fig. 6B-E), while when GOP was administrated, the values of IL-5, IL-12p70 and TNF-α returned to normal levels. Effects of low doses of GOP showed a slight trend on IL-6 compared to control (*p* < 0.10). Concentrations of other cytokines studied did not show significant changes (data not shown).

When treated with GOP and whey protein, the reduction of small intestinal SIgA levels in irradiated mice was significantly vanished and finally returned to a normal level (Fig. 6F).

As shown in Fig. 6G, the increases in serum of IgA, IgG2a, IgG2b, IgG3 and IgM concentrations were observed in the irradiated mice, while the treatment with GOP resulted in a normalization of all the Ig’s studied. When compared with the whey protein results, the effect of GOP was ameliorated.

### GOP treatment is resistant to irradiation-induced cell apoptosis, inhibits the expression of apoptosis proteins and improves the expression of tight junction proteins

**Figure 7:**
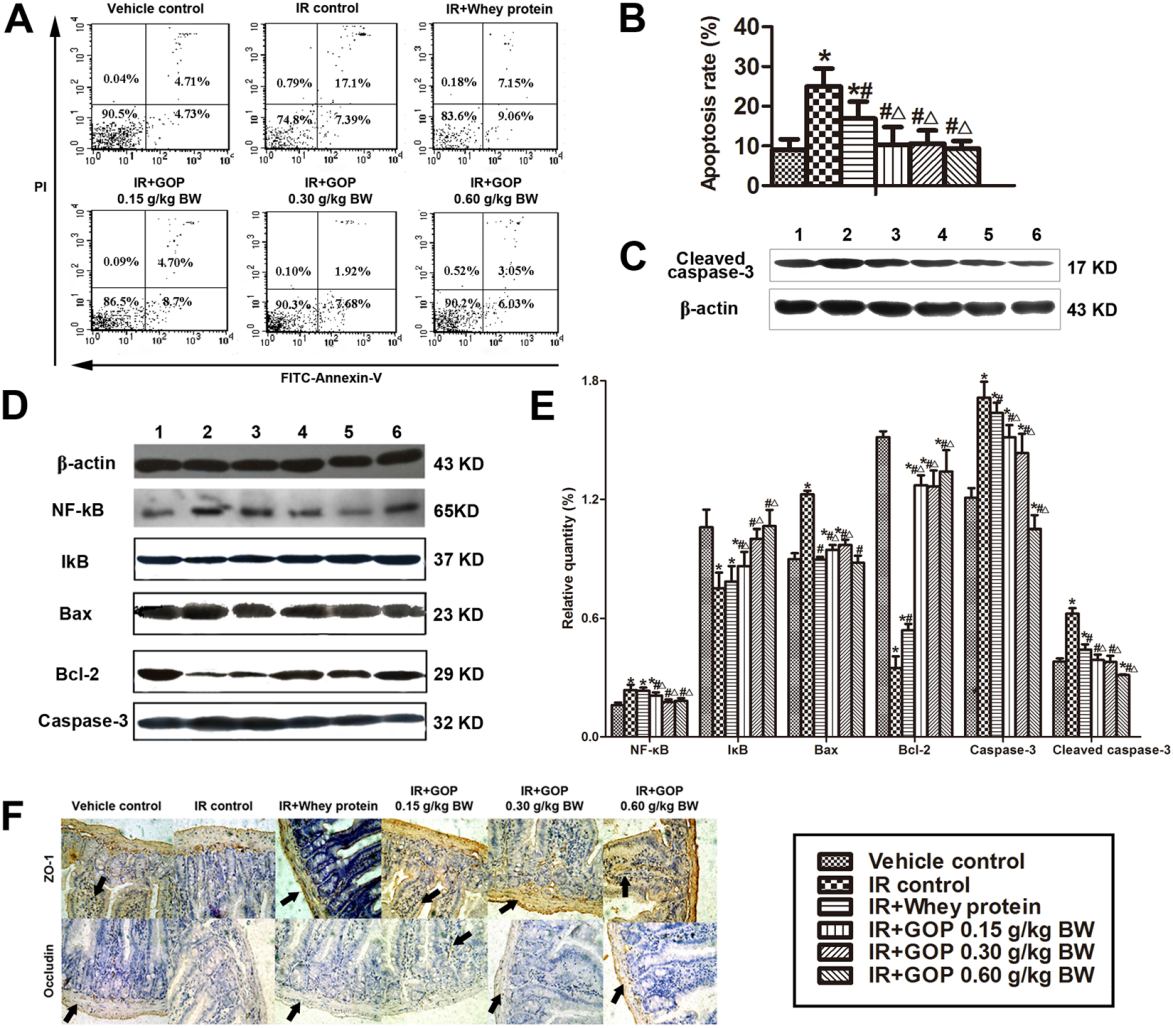
GOP administration is resistant to cell apoptosis, inhibits apoptosis proteins and improves tight junction proteins expression of irradiated mice. (**A**) Cell apoptosis was analyzed by Annexin V-FITC/PI double staining via flow cytometry. (**B**,**C**) Apoptosis-related protein levels were analyzed by Western blot; 1, vehicle control, 2, IR control, 3, IR + Whey protein, 4, IR + GOP 0.15 g/kg BW, 5, IR + GOP 0.30 g/kg BW, 6, IR + GOP 0.60 g/kg BW. (**D**) The apoptotic cells (Annexin V + /PI + ) were quantified by FITC Annexin V Apoptosis Detection Kit with PI. (**E**) The relative quantity of apoptosis-related protein levels was analyzed by Image-Pro Plus software. (**F**) Tight junction protein levels were determined by immunohistochemistry. Values represented the mean ± SD (n = 12 per group) from a single experiment, which was analyzed by ANOVA test, and followed by least-significant difference for posthoc test between multiple groups. All the measurements were done in duplicates. *p < 0.05 versus vehicle control group, ^#^*p* < 0.05 versus IR control group, and ^△^*p* < 0.05 versus IR + whey protein group.

To clarify the mechanisms of the protective roles of GOP in irradiated mice, we analyzed the apoptosis rates of splenocytes, the expression of apoptosis-related proteins in the spleen and the tight junction proteins in the intestine (Fig. 7A–E). The administration of GOP resulted in a reduction of the irradiation-induced apoptosis rate, which was comparable to the values observed in the vehicle control. The results obtained using GOP were more robust than the ones obtained using the whey protein (Fig. 7A,B). In addition, we observed that irradiations were linked to a significant increase in the expression of NF-κB, Bax, Caspase-3 and Cleaved caspase-3 and a significant decrease in the expression of IκB and Bcl-2. Importantly, the treatment of GOP on irradiated mice markedly blocked these changes, and the effects observed by using GOP were better than the ones using the whey protein (Fig. 7C,D).

As a key structure of intercellular space, tight junction plays a major role in regulating the paracellular permeability of the epithelium, which resides near the apical surface of adjacent epithelial cells. The tight junctions consist of transmembrane proteins such as occludins, claudins, junctional adhesion molecules, and intracellular scaffold proteins [such as zonula occluden (ZO) proteins]^11^. From the immunohistochemical results (Fig. 7E), we found that tight junction proteins (ZO-1 and Occludin) in the vehicle group were expressed in the cytomembrane of epithelial cells, and most commonly in the spinous and granular layers, whereas irradiation significantly reduced the expression of ZO-1 and Occludin. In accordance with the previous results, GOP administration prevented the reduction of these proteins while reverting the expression to a normal level, in both cytomembrane and cytoplasm of spinous and granular layers in the mucosa.

## Discussion

As a human colonic epithelial cell line, Caco-2 retains all the features of differentiated intestinal epithelial cells such as defined brush borders and formation of tight junctions, which has been used in many studies to assess the different disorders that can affect the intestinal epithelial barrier^12,13^. In this study, we examined the effects of GOP on the epithelial barrier function of irradiated Caco-2 cell monolayers cultured on trans-wells membranes. We found that GOP had a beneficial effect on irradiation-induced decreased TEER and hyperpermeability to FITC-dextran. In support of these results, we performed *in vivo* studies to confirm the effects and further elucidate the mechanism.

Previous studies demonstrated that proton whole-body irradiation derived from a ^60^Co gamma-ray source could result in an acute immunodepression, with the greatest immunodepression observed at 4 days postexposure^14^. In this study, we tested the protective effects of the GOP by using a model of BALB/c mice treated by proton irradiation to investigate whether GOP possesses immunomodulatory and intestinal damage repair effects on irradiated animals. In order to ensure reproducibility of our results and to stay in line with previous studies, we conducted our experiments taking the four days after irradiation time window as a basic parameter, and try to establish a way to maximize protection effects of GOP on normal tissues during and after proton-radiation exposure. Our results demonstrated that irradiation led to a significant reduction in the proliferation response of splenic lymphocyte in response to ConA and LPS and the productions of IL-6 and IL-12 in culture from splenocytes, indicating that irradiation inhibited the activity and the proliferation of splenocytes. Meanwhile, we found CD3^+^ and CD4^+^ were dramatically increased in both spleen and mesenteric lymph nodes by irradiation. As reported from Kajioka *et al*.^14^, lymphocyte populations differ in radiosensitivity, where B cells were most sensitive, T (CD3^+^) cells were moderately sensitive, and natural killer (NK1.1^+^) cells resulted in the most resistant; in this study, comparison of the T lymphocyte subpopulations showed that CD4^+^ (T helper/inducer cells) were more radiosensitive than CD8^+^ (T cytotoxic/suppressor cells), especially in mesenteric lymph nodes. Williams *et al*.^15^ monitored the recovery of T lymphocyte subsets in mice were exposed to a 6.5-Gy radiation dose and found the relative radioresistance of CD4^+^ cells resulted in a selective enrichment of these cells among the surviving thymocytes and splenic lymphocytes. This relative enrichment of CD4^+^ cells became even more prominent 7 days after irradiation even though the atrophy of the organs was greatest. This could support our result that the indices of spleen and thymus in irradiated mice even treated with GOP were still significantly lower than the control group in our study even though the percentages of CD3^+^ and CD4^+^ were much higher than the irradiation group and control group.

Intestinal injury is a very common complication of radiotherapy, with the small intestine being one of the most radiosensitive organs. In a study by Monti *et al*., intestinal mucosal injury was the main predictor of survival in patients exposed to high doses of radiation^16^. Irradiation therapy usually leads to intestinal architectural disorganization, including inflammatory mononuclear cell infiltration, eosinophilic necrosis, villitis and villous height decrease^17,18^, which was present in the current study. Moreover, with lymphocytes being highly sensitive to ionizing radiation, it induces the release of danger associated molecular patterns (DAMPs), which are an endogenous immune adjuvant that further stimulates the dendritic cells (DCs), inducing them to provide co-stimulatory signals to naïve T-cells, resulting in cross-priming of cytotoxic T-lymphocytes (CTLs)^19^. Furthermore, through the induction of chemokines, irradiation increases recruitment of effector T-cells to irradiation site^19^. Despite of the radiation-induced impairment of both the cytokine homeostasis profile with T-cell lineages and T helper/ T cytotoxic homeostasis, GOP administration facilitated recruitment of CD3^+^, CD4^+^ and CD8^+^ resulting in restored capacity and significant improvement in T-cell reconstitution, along with amelioration of cytokine homeostasis.

Immunomodulators have been considered as one of the most promising alternatives to classical drug treatment to alter host immune dysfunction^20^. Although the mechanism underlying the immunomodulatory effects of food-derived bioactive peptides remains uncertain, there are several studies showing that they may enhance lymphocyte proliferation and antibody synthesis^21^, macrophages function^22,23^, humoral immune response, CD4^+^ T helper percentage, cytokines^20^ and NK activity^24^. Derived from ginseng (*Panax ginseng* C. A. Mey.) by enzymatic hydrolysis, GOP is mainly considered small molecule oligopeptides. Importantly, we have demonstrated that mice treated with GOP were characterized by a reduction in intestinal permeability (high FITC-dextran and DAO concentrations in plasma) and metabolic endotoxaemia (high LPS concentration in plasma) induced by irradiation, which was proposed to be associated with the disruption of the crucial tight junction proteins, ZO-1 and occluding^25^. DAO is a highly active enzyme existing within the intestinal villus epithelial cells in humans and mammals in general, which is an important indicator of intestinal integrity and barrier function. Moreover, the villus height and crypt depth also reflect the extent of the intestinal mucosal damage. When the intestinal barrier is damaged, there is an increase of DAO in the blood, as well as endotoxin (LPS). The leakage of LPS derived from intestinal microflora into portal blood is a well-established mechanism of metabolic endotoxemia triggering inflammation and oxidative stress^25,26^. We found that by lowering systemic inflammation using GOP was accompanied with a marked reduction in plasma LPS level as well as the indices of oxidative and inflammatory stress[ie, decrease in circulating cytokines (IL-1α, IL-5, IL-12 and TNF-α) and immunoglobulins (IgA, IgG, and IgM) induced by irradiation]. We, therefore, proposed that GOP could decrease endotoxemia-induced inflammation and metabolic disorders by increasing the activities of the antioxidant enzymes, such as SOD and GSH-Px. Song *et al*.^27^ also suggested that ginseng may contribute to the protective activity against radiation through the induction of antioxidant enzymes such as SOD, catalase, and GSH-Px. The effects of GOP resulted also in the activity of scavenging free radicals and decreasing the production of lipid peroxidation (MDA), in order to maintain the cell membrane integrity and avoid intestinal mucosa damage, therefore decreasing intestinal permeability. Zhang *et al*.^28^ concluded that the water-soluble extract (such as carbohydrate and protein) of whole ginseng exhibited the best protection against γ radiation, which were similar to the results from Kim *et al*.^29^ by reporting that the whole extract and its fractions of the whole ginseng decreased the apoptosis of jejunal crypt cells and increased endogenous spleen colony formation in irradiated mice. Among the involved mechanisms, excessive TNF-α production induced by irradiation presumably expand local or systemic inflammation, which could trigger a disturbance of both junctional proteins and the mucous barrier functions^30,31^. Irradiated mice treated with GOP were characterized by lower serum IL-1α and TNF-α concentrations, well-known to accelerate tight junction disruption^32–34^. IL-6 is one of the most important pro-inflammatory cytokines but it has also been considered to have an anti-inflammatory role for its ability to induce IL-1 and TNF-α antagonists^35,36^. Different from other pro-inflammatory cytokines, we observed that irradiation caused a significant decrease in serum IL-6 concentration, whereas GOP administration increased the IL-6 level and returned it to the normal level. Song *et al*.^27^ also reported that ginseng was able to simultaneously induce a variety of radioprotective cytokines (such as IL-1β and IL-6) and potentially modulate the balance between Th1 and Th2 cytokines and restore the normal cytokine balance broken down by radiation. Increased IL-6 production may have also beneficial health effects, since IL-6 possesses several anti-inflammatory activities, such as down-regulation of LPS-induced TNF-α mRNA expression. Enhanced mucosal SIgA response is considered to reduce allergic and inflammatory responses and inhibit leaking of antigens in mucosa to the gastrointestinal tract and associated lymphoid tissue (GALT)^37^. In general, the protective effects were better than whey protein, therefore, we postulated that the effects may be attributed to GOP, but not to the increase of protein intake.

Among the putative mechanisms, inflammatory responses are mediated by the activation of nuclear factor κB (NF-κB), which is normally combined with inhibitor κB (IκB) in the cytosol to prevent its entrance into the nucleus^38,39^. Pro-inflammatory stimuli could result in the phosphorylation of IκB which in turn release NF-κB, that translocates into the nucleus where it induces the transcription of pro-inflammatory cytokines and enzymes generating reactive oxygen species (ROS)^40^. NF-κB signaling pathway provides a highly attractive target for the therapeutic development, and more than 700 inhibitors of the NF-κB activation pathway were reported, including peptides, antioxidants, small molecules, microbial and viral proteins, small RNA/DNA, and engineered dominant-negative or constitutively active polypeptides^41^.The canonical NF-κB activation pathway—triggered by TNF-α, IL-1 or byproducts of bacterial and viral infections (such as LPS and double-stranded RNA)—is dependent on the IKKβ catalytic subunit and is accomplished through IκB phosphorylation and ubiquitin-dependent degradation^42^. The activation of NF-κB by many stimuli depends on the phosphorylation of IκBs at N-terminal sites by the IKK complex, while the mechanism of NF-κB activation by ultraviolet (UV) radiation, γ rays or ionizing radiation (IR) involves the IKK-independent phosphorylation of IκBα at a cluster of C-terminal sites^41,43^. Previous studies also reported that mitochondrial electron transport inhibitors that suppress reactive oxygen intermediate production and overexpression of antioxidizing enzymes (e.g., manganese superoxide dismutase and catalase) can block TNFα-induced NF-κB activation^44–46^.

Although the ways in which GOPs block NF-κB activation remain unclear, we speculate that GOP pre-administration might inhibit over-expression TNFα-induced NF-κB activation by ultraviolet (UV) radiation; on the other hand, we think that GOPs might act like antioxidants, which have been suggested to inhibit NF-κB activation by scavenging reactive oxygen intermediates that act as signaling molecules to activate the NF-κB pathway and by directly inhibiting IKK kinase activity by modifying critical Cys residues in the IKK kinase activation loop^47,48^.

Subsequently, as an important transcription factor, NF-κB not only controls inflammation but also acts on apoptosis. NF-κB induces a variety of anti-apoptotic factors, including the inhibitors of caspase activation and action, anti-apoptotic Bcl2 family members and inhibitors of Jnk activation, which can prevent TNF-α–induced apoptosis^42^. Bax and Bcl-2 are the main apoptosis-related proteins belonging to the Bcl-2 super-family, which regulate the mitochondrial membrane permeability. The activity of these two proteins is intimately related, and the interaction of both proteins in forming a heterodimer, lead to the maintenance of the mitochondrial membrane integrity, by blocking the pore-forming activity of Bax, therefore preventing the release of cytochrome c (Cyt c) from the mitochondria, hence reducing the oxidative stress damages^40,49,50^. Caspase-3 is another apoptosis-induced protein. Caspases posses a central role for most apoptosis inductions^51^. Bcl-2 is believed to prevent apoptosis through regulating the mitochondrial pore permeability transition and by inhibiting caspases via an intermediate^52^. We propose that the ability of GOP administration to block the apoptosis is related to the decreased expression of Bax and Caspase-3 and increased expression of Bcl-2.

The main functional components of whey protein are β-lactoglobulin, α-lactalbumin, bovine serum albumin and immunoglobulin, and glycomacropeptide, while the minor proteins are lactoperoxidase, lactoferrin, β2-microglobulin, lysozyme, insulin-like growth factor, γ-globulins, etc.^53^. From several trials in human and animals it has been shown that whey protein is easily digested and promptly absorbed, allowing this protein to have a high bio-availability and multi-functions, including anti-inflammatory, immune-enhancement, antioxidant, and anabolic effects^54^. Several studies have indicated that whey proteins exert significant protective effects against oxidant stress imbalances associated with many chronic and acute disease conditions such as diabetes, heart disease, inflammatory bowel disease (IBD) and cancer^55,56^. Using whey protein as a control for our experiments with GOP, we can exclude the possibility to obtain false positive results that may be caused by an increased protein intake. In the present study, we found that GOP possessed better radioprotective effects compared with the ones obtained by using whey protein, therefore we could conclude that the radioprotective effects were due to oligopeptides, and not protein intake. In general, protein digestion and absorption in humans depends on initial enzymatic hydrolysis in the stomach and proximal small intestine. The hydrolytic products include oligopeptides and amino acids that ultimately undergo small intestinal uptake into the portal vein. As for peptide absorption, it has been well concluded that there are two major mechanisms involved: the hydrolysis of peptides by brush border enzymes with subsequent uptake of the liberated amino acids by specific amino acid transport systems, and the uptake of peptides by mechanisms independent of the specific amino acid entry mechanisms, followed by intracellular hydrolysis^57^. Silk *et al*.^57^ reviewed that oligopeptides could not only be transported intact into the mucosa cell by special peptide uptake systems in normal human subjects, but also mucosal uptake of small peptides has an important or potentially major role in protein absorption, which is even more efficient than free amino acids up-take^57^. However, he also summarized that with the possible exception of glycyl-glycine and hydroxyproline peptides, oligopeptides do not pass into the portal circulation^57^. Nevertheless, Gestin *et al*.^58^ concluded that arginine-rich peptides have presented concentration-dependent mechanisms, being taken up either by membrane destabilization or clathrin-mediated endocytosis. The absorption and metabolism of GOPs is not very clear, therefore, for the further study, we could use isotopes or fluorescent substance to label GOPs, and check the concentration of isotopes or fluorescent substance in the blood and organs of the mice after the oral administration, in order to trace the GOPs and explore the mechanism.

In conclusion, the administration of GOP protects intestine and immune system from radiation-induced injury and dysfunction, including reducing intestinal permeability and local and systemic inflammation, increasing antioxidant ability, promoting proliferation response, and decreasing the expression of apoptosis-related proteins. Our findings were supported by different experimental approaches and represent a promising therapeutic application of GOP in gastrointestinal or immunosuppressive disease.

## Methods

### Preparation and identification of GOP

Through enzymatic hydrolysis, GOP was prepared from the root of *Jilin* ginseng (*Panax ginseng* C. A. Mey.). After initial cleansing, mincing, and homogenization in distilled water; it was added into complex protease (3000 U/g protein) at 40 °C and pH of 8.0 for 3 hours. Followed by centrifugation, it was separated by ceramic membrane to further purify it. This was followed by series of subsequent procedures, namely nanofiltration, cryoconcentration under vacuum at 70 °C, decolorization, purification, and spray drying, until GOP powder was finally obtained.

The GOP powder was then further purified by High-Performance Liquid Chromatography (HPLC, Water Corp., Milford, MA, USA) and determined by LDI-1700 matrix-assisted laser desorption ionisation time-of-flight mass spectrometry (MALDI-TOF-MS, Linear Scientific Inc., Reno, NV, USA). Then, we measured the molecular weight distribution along with amino acid composition analysis using the automatic amino acid analyzer (H835-50, Hitachi, Tokyo, Japan). Using the HPLC to estimate the amount of free amino acids, the small molecule oligopeptides (defined as relative molecular weight was between 180 and 1000) in GOP was 95.42%, and free amino acids comprised 3.94%. The amino acids composition analysis demonstrated GOP samples to be rich in Arg > Pro > Asp > Ala > Glu (Table 2).

**Table 2:** The amino acid composition of ginseng oligopeptides.

Amino acid	Contents (g/100 g)
Aspartic acid	0.19
Glutamic acid	0.12
Serine	0.02
Histidine	0.06
Glycine	0.02
Threonine	0.05
Arginine	2.26
Alanine	0.13
Tyrosine	0.09
Cysteine	0.01
Valine	0.06
Methionine	0.02
Phenylalanine	0.09
Isoleucine	0.04
Leucine	0.08
Lysine	0.06
Proline	0.65

### Cell culture

Human colorectal adenocarcinoma cells line (Caco-2) cells were cultured using Dulbecco’s Modified Eagle medium (DMEM) in addition to the following: 10% fetal bovine serum, 100 units/ml penicillin and 100 μg/ml streptomycin in a humidified incubator at 37 C with an atmosphere of 5% CO_2_; with change of media every other day. Caco-2 cells (passages 26–40) were seeded at 1 × 10^5^ cells/ml on polycarbonate 12-well Transwells® (Corning Costar Corporation, Cambridge, MA) (0.4 μm mean pore size) and used for transport experiments 21–28 days after seeding.

### Irradiation setup and TEER measurement

The trans-epithelial electrical resistance (TEER) of Caco-2 monolayer formation was assessed using EVOM^2^ Epithelial Voltmeter (World Precision Instruments) after 19–21 days in culture. It has established in the literature that Caco-2 cells can be used for permeabilization TEER measurements only when TEER values range between 300–600 Ω/cm^2^.

Cells were treated with whey protein 150 μg/ml whey protein, in addition to different concentrations of GOP (50, 150 and 200 μg/ml) for 24 hours. After the initial assessment of TEER values, cells were subsequently irradiated with 2 Gy X-ray, with a dose rate of 1.14 Gy/min at room temperature, using FAQ RS-2000 Irradiator (Rad Source Technologies) in Dana-Farber Cancer Institute (Boston, USA). The environmental/procedural conditions of the sham-irradiated cells were similar to the irradiated cells. TEER measurements was performed initially before the irradiation and hourly for the first 6 hours post-irradiation, then every 3 hours till 48 hours, in presence/absence of different concentrations of GOP or whey protein.

### Caco-2 monolayer permeability

Caco-2 cell monolayers permeability to 4000 Da fluorescein isothiocyanate-dextran (FITC-dextran, Sigma-Aldrich) was assessed upon incubation with/without different concentrations of GOP and whey protein. After adding 200 μl of basal medium (without phenol red) containing 20 μg of FITC-dextran (100 μg/ml) to the apical compartment of the trans-wells, the cells were incubated for 4 hours. Then, the media were collected subsequently from basal compartments at 30, 60, 90, 120, 180 and 240 minutes. Finally, paracellular permeability to FITC-dextran was evaluated through measuring the fluorescence intensity at 485 nm/525 nm using a fluorescence multi-plate reader (Spectra Max i3x, Molecular Devices).

### Animal studies

Eight weeks old female BALB/c mice were obtained from the Animal Service of Health Science Center, Peking University. The mice were group-housed in a temperature-controlled environment with a 12 hr light/dark cycle, fed the AIN-93 diet, and diet and water were provided *ad libitum*. All animal studies were performed according to the National Institutes of Health guidelines for the Care and Use of Laboratory Animals (NIH publication no. 85-23, revised 1996) and the protocols were approved by the Peking University Animal Research Committee.

### ^60^Co-gamma-ray irradiation

The mice were placed individually in a close-fitting Perspex box (3 × 3 × 11 cm), underwent whole-body irradiation of a ^60^Co-gamma ray in the irradiation center in Peking University, China. The irradiated mice were given a single dose of 3.5 Gy with a dose rate of 0.79 Gy/min.

### GOP treatment and Induction of immune dysfunction and intestinal injury

Three experiments were performed to assess the GOP effects on irradiation-induced gastrointestinal injury, oxidative damage and immune dysfunction. In every experiment, the animals were randomly divided into six groups: (1) vehicle control group: where the vehicle was administered intragastrically into the mice; (2) irradiation (IR) control group: mice were also intragastrically administered with the vehicle; (3) IR + whey protein group: mice were intragastrically administered with 0.30 g/kg BW whey protein; and (4) IR + GOP groups: mice were intragastrically administered with 0.15, 0.30 and 0.60 g/kg body weight (g/kg BW) GOP. All the mice recieved 1 mL/100 g BW, with the mice and food intake being weighed every one week.

Except for the vehicle control group, all mice received a single dose of 3.5 Gy with ^60^Co-gamma-ray exposure on the 30th day. At four days after irradiation, blood samples were collected from ophthalmic venous plexus followed by mice scarification through cervical dislocation. The spleen, thymus, and liver were then removed, weighed, and the organ indexes were calculated. The intestine (duodenum, jejunum, and ileum) was removed to undergo histopathology, immunohistochemistry, Western blotting and ELISA analyses.

### Histopathology analysis and crypt and villus morphology

Following fixation and hydration, small intestinal tissues were embedded in paraffin to produce four cross-sections per mouse. Then slides were stained with hemotoxylin and eosin (H & E) for histopathology analysis by light microscopy. Around 20-40 separate measures of villus length and crypt depth per mouse were obtained to calculate the mean length and depth for each mouse^59^.

### Intestinal permeability *in vivo*

In order to evaluate the extent of intestinal injury, *in vivo* intestinal permeability measurement was assessed at one and three days following irradiation, using 4000 Da FITC-dextran (Sigma-Aldrich, St. Louis, MO) as described^34,60^. Briefly, mice fasting for 6 h were gavaged with FITC-dextran (60 mg/100 g BW, 60 mg/mL). After 4 h, 120 μL of blood was collected from ophthalmic venous plexus, centrifuged at 4 °C, 12,000 *g* for 3 min. After plasma dilution in an equal volume of PBS (pH 7.4), standard curves were obtained by diluting the FITC-dextran in the PBS-diluted plasma. FITC concentration in the plasma was then measured with FlexStation 2 fluorescence microplate reader (Molecular Devices, Sunnyvale, CA) at an excitation wavelength of 485 nm and an emission wavelength of 520 nm.

### ELISA and Multiplex sandwich immunoassays for biochemical assays

Superoxide dismutase (SOD), glutathione peroxidase (GSH-Px) and malondialdehyde (MDA) levels in serum and liver; and diamine oxidase (DAO) and endotoxin (lipopolysaccharide, LPS) levels in plasma were measured by the mouse ELISA kits purchased from Andygene Co. (Richardson, Texas, USA). The enzyme unit of SOD and GSH-Px is defined as the number of micromoles of reduced substrate per minutes at 37 °C in 1 mL of supernatants under standard assay conditions.

Serum levels of cytokines and immunoglobulins were determined from the samples using the multiplex sandwich immunoassays with the Milliplex Mouse 11 Cytokine/ 5 immunoglobulins Premixed Kit (Millipore; Billerica, MA) and established protocols in accordance with manufacturer’s instructions. The 11 cytokines included interleukin (IL)-1α, IL-2, IL-4, IL-5, IL-6, IL-10, IL-12p70, IL-17, interferon (IFN)-γ, tumor necrosis factor (TNF)-α and granulocyte-macrophage colony-stimulating factor (GM-CSF). The 5-immunoglobulins panel comprised IgA, IgM, IgG1, IgG2a, IgG2b, and IgG3. A five-parameter model was used to calculate final concentrations and values are expressed in pg/mL.

### Lymphocyte proliferation assay induced by Con A and LPS

Spleens of the mice were sacrificed under aseptic conditions, digested to obtain a single-cell suspension, then seeded in 24-well plates (1 mL/well) at a concentration of 5 × 10^6^ cells/mL with/without (control wells) 75 μL/well Con A or LPS as a T- or B-cell stimulant in triplicate. After 68 h incubation at 37 °C under humidified 5% CO_2_–95% air condition, 0.7 mL of supernatant per well was removed, then 0.7 mL of RPMI-1640 without FBS and 50 μL of MTT (5 mg/mL) were added to each well, and the cells were incubated for 4 h at 37 °C under humidified 5% CO_2_–95% air condition. Thereafter, 1 mL of 3% (w/w) SDS solution was mixed with insoluble purple formazan in each well resulting in colored solution that was measured using an ELISA reader at the absorbance of 570 nm (Bio-Rad, Hercules, CA). Proliferation capacity was represented by the difference of absorbance with/without ConA or LPS.

### Flow cytometry analysis for T lymphocyte subpopulations

For T lymphocyte subpopulations assay, single-cell suspension of splenocytes and mesenteric lymph nodes was adjusted to a concentration of 1 × 10^6^ cells/mL. T-cell specific markers were stained using PerCP-Cy5.5-conjugated anti-mouse CD3, FITC-conjugated anti-mouse CD4, Pacific Blue-conjugated anti-mouse CD8a, PE-conjugated anti-mouse CD25, PE/Cy7-conjugated anti-mouse NK-1.1 (only in splenocyte) and Alexa Fluor 647-conjugated anti-mouse foxP3 (only in mesenteric lymph node lymphocytes) (BioLegend, San Diego, CA, USA). We then conducted fluorescence activated cell sorting (FACS) and data analysis using a Beckman Gallios™ flow cytometer (Beckman Coulter, Pasadena, CA, USA), after adjusting the instrument settings using the control cells stained with isotype-matched antibodies.

### Small intestinal secreted immunoglobulin

Following the excision and weighing of the mice’s organs, their small intestine was removed from the duodenum to the terminal ileum, flushed with 2 mL cold calcium- and magnesium-free Hank’s balanced salt solution to remove intestinal contents^61^. Washing fluids were collected and stored at −80 °C for the analysis of intestinal secreted immunoglobulin (S-IgA) levels by ELISA kit purchased from Andygene Co. (Richardson, Texas, USA)^62^.

### Splenocyte culture for IL-6 and IL-12 assay

The concentration of splenocytes suspension was adjusted to of 3 × 10^5^ cells/mL. After 64 h, IL-6 and IL-12 concentrations were measured in the culture supernatants using ELISA kits according to the manufacturer’s protocol.

### Apoptosis assay

5 μL of FITC Annexin V and 10 μL of propidium iodide (PI) were added to the collected splenocytes following their resuspesion in 500 μL of Annexin V Binding buffer provided with the FITC Annexin V Apoptosis Detection Kit with PI (BioLegend, San Diego, CA, USA) at the density of 1 × 10^6^/mL. The cells were incubated for 15 min at room temperature in the dark, stained, and then transferred to Falcon flow tubes for analysis using FACSCalibur Flow Cytometer (BD Bioscience, New Jersey, USA).

### Immunohistochemistry

Paraffin-embedded intestinal tissue sections were used for immunohistochemistry. Tight junction proteins ZO-1 and Occludin expression was detected using rabbit anti-ZO-1 (1:1000, Abcam, Cambridge, MA, USA) and rabbit anti-Occludin antibodies (1:50, Abcam, Cambridge, MA, USA), respectively. Thereafter, the sections were immunostained with goat anti-rabbit IgG conjugated to horseradish peroxidase (HRP) using a DAB kit (Zhongshan Goldenbridge, Beijing, China).

T-cell markers CD3, CD4, CD8a, and CD25 were detected using mouse anti-CD3, rat anti-CD4, mouse anti-CD8a and mouse anti-CD25 (1:250, Santa Cruz Biotechnologies, Dallas, TX, USA), respectively. For the secondary antibody, we utilized goat anti-rabbit IgG conjugated to horseradish peroxidase (HRP) using a DAB kit (Vector Laboratories, Inc., Burlingame, CA, USA).

### Western blotting analysis

Different protein concentrations were measured by a Bradford assay. Proteins were initially separated on SDS-polyacrylamide gels and electrotransferred to polyvinylidene fluoride (PVDF) membranes (Millipore, Billerica, MA, USA). Then, membranes were blocked for 2 h in Tris-buffered saline containing 5% nonfat milk and 0.1% Tween 20 at room temperature, followed by incubation with a primary antibody against β-actin (1:5000; Santa cruz, Dallas, Texas, USA), NF-κB (1:1000; Abcam, Cambridge, MA, USA), IκB (1:2000; Abcam, Cambridge, MA, USA), bax (1:1000; Abcam, Cambridge, MA, USA), bcl-2 (1:100; Abcam, Cambridge, MA, USA) and caspase-3 (1:100; Abcam, Cambridge, MA, USA) overnight at 4 °C. Secondary-antibody incubation with peroxidase-conjugated anti-rabbit antibody (1:4000; Santa Cruz, Dallas, Texas, USA) was performed for 2 h at room temperature. Finally, chemiluminescence reaction and Hyperfilm ECL were utilized for visualization of the protein bands, with their relative quantitative analysis done using Image-Pro Plus analysis software (Media Cybernetics, Silver Spring, MD, US).

### Statistical analysis

Data were reported as mean ± standard deviation (SD), analyzed by one-way analysis of variance (ANOVA) test, then multiple comparisons of least-significant difference (equal variances assumed) or Dunnett’s T3-test (equal variances not assumed) were conducted to detect any difference of parametric samples among the groups. Final analyses of Luminex data for cytokines considered only data that fell within the detection limits of the Luminex assay. Concentrations obtained below the sensitivity limit of detection (LOD) of the method were calculated as LOD/2 for statistical comparisons as reported in previous studies^63–65^. If the raw or log-transformed data did not meet the criteria for normality assumption, the nonparametric Kruskal-Wallis, and Mann-Whitney test was used to determine statistical differences, along with Bonferroni correction to account for multiple comparisons. Statistical significance was set at *p*-value < 0.05. All statistical analyses were carried out using IBM SPSS Statistics version 20.0 and GraphPad Prism 5 Software.
